# Prevalance and clinical characteristics of gingival recession in Greek young adults: A cross‐sectional study

**DOI:** 10.1002/cre2.427

**Published:** 2021-05-03

**Authors:** Ioannis Fragkioudakis, Dimitra Tassou, Maria Sideri, Ioannis Vouros

**Affiliations:** ^1^ Department of Preventive Dentistry, Periodontology and Implant Biology, Faculty of Dentistry Aristotle University of Thessaloniki Thessaloniki Greece

**Keywords:** gingival recession, prevalence, risk factors, young adults

## Abstract

**Objective:**

The current cross‐sectional study aimed to investigate the prevalence of gingival recession (REC) in a sample of young individuals. In addition, the association with several risk factors was examined.

**Materials & methods:**

A 104 subjects, aged 18–30 years old, were randomly enrolled in the study. Participants were requested to fill in a simple structured questionnaire in order to provide information on dental hygiene habits, educational level, smoking and history of orthodontic treatment. Afterwards, all the included individuals were subjected to a thorough clinical periodontal examination including gingival recession (REC), gingival biotype, plaque levels and gingival bleeding index (GBI) assessment.

**Results:**

The mean prevalence of REC in the studied population surpassed 50% with an equal distribution among females and males. The most common teeth associated with REC were the lower left canine and left 1st premolar. Among the examined variables, only the bleeding index was found to be associated with the presence of REC.

**Conclusions:**

Gingival recession was a prevalent condition among young individuals. Gingival inflammation was found to be the most significant factor affecting the incidence of REC.

## 1 INTRODUCTION

Gingival recession (REC) is defined as the apical migration of the gingival margin concerning the cemento‐enamel junction (CEJ) (Pini Prato, 1999). Gingival recession, although not associated with increased tooth loss, is considered an esthetic issue in many patients and is often associated with the occurrence of dentin hypersensitivity and carious/non‐carious cervical lesions on the exposed root surface. (Cortellini & Bissada, 2018). Furthermore, REC has been reported as a factor negatively influencing the Oral Health‐Related Quality of life (OHQol) as it seems to affect functional limitation, physical pain, psychological discomfort, physical and psychological disabilities (Wagner et al., 2016).

Several risk factors have been implicated in the occurrence of gingival recession, such as the presence of a thin biotype, lack of attached gingiva, presence of bone deficiencies, tooth malposition, and high frenum or muscle attachment. (Moawia & Cohen, 2003; Susin et al., 2004; Zweers et al., 2014). In addition, iatrogenic factors as the presence of faulty restorative cervical margins and previous orthodontic therapy, have been proposed as potential predisposing agents of REC (Kim & Neiva, 2015). However, the main etiological factors associated with the occurrence of gingival recession are inflammatory periodontal conditions and mechanical trauma (Löe et al., 1992).

The prevalence of REC varies among different populations and age cohorts. Several studies have demonstrated a prevalence of 50%–99.7%, increasing with age in Brazilian and Turkish populations. (Albandar & Kingman, 1999; Susin et al., 2004; Toker & Ozdemir, 2009). Data from NHANESS suggest that REC of ≥1 mm reaches 58% in an American population over 30 years old (Albandar & Kingman, 1999). Despite the frequent occurrence, data on the prevalence, however, are still scarce, since most studies report on a limited number of individuals and in specific geographic locations. Data on REC in the Greek population are also limited (Chrysanthakopoulos, 2014). Thus, this study aimed to assess the prevalence of REC in a sample of young Greek Adults, and its association with a number of risk factors.

## 2 MATERIALS AND METHOD

### 2.1 Study design‐study sample

The study was designed as a cross‐sectional study. The participants were either patients, treated in the undergraduate Periodontology Clinics of the Dental School, Aristotle University of Thessaloniki or dental students. The enrollment of the participants in the study was based on the following:

Inclusion criteria:

- Age 18–30 years old;
- Fully dentate participants;
- Periodontal health or gingivitis.

The exclusion criteria were set as:

- Presence of periodontal pockets with depth ≥5 mm;
- Insufficient restorations or prostheses.
- Systemic disease affecting the periodontal tissues (e.g., bleeding disorders, diabetes mellitus etc.)
- Patients under medication associated with gingival enlargement (e.g., calcium channel blockers, immunosuppressants or anticonvulsants).
- Third molars were also excluded from the study.

All participants received a full oral examination and afterwards were asked to answer a simple structured questionnaire. The study was conducted between September 2016 and June 2019.

### 2.2 Clinical examination

Each subject was examined for the presence of gingival recession (REC). REC was defined as the distance between the gingival margin and the CEJ in the mid‐buccal surface of each tooth. Other factors examined and associated with REC were:

- Plaque index (PI) (O'Leary et al., 1972);
- Gingival bleeding index (GBI) (Carter & Barnes, 1974) (presence or absence of bleeding, examined in six surfaces per tooth);
- The periodontal biotype evaluation was based on measurements of the keratinized gingival thickness (GT). The GT was measured using an endodontic file K No 10 with a stop ring. The file was inserted into the gingiva at 2 mm apically to the gingival margin of the right central incisor until it reached the alveolar bone. In order to achieve a sufficient fit of the K file, the central hole of the stop ring was avoided and the penetration was performed adjacent to it. The GT was defined as the distance between the file peak and the apical margin of the stop ring. The biotype was classified as thick if GT was ≥1 mm and thin if GT was <1 mm (Kan et al., 2010).

Clinical examinations were performed by two trained examiners (I.F., D.T.). All measurements were conducted with a @Hu‐Friedy CP‐15, #30 periodontal probe. Before the initiation of the study, a calibration session was conducted on ten patients. Measurement of gingival recession was performed and repeated twice within 1 week by both examiners. The resulting inter‐examiner intraclass correlation coefficient was 0.896 (95%CI, 0.767–0.973). The intra‐examiner intraclass correlation coefficients were 0.865 (95%CI, 0.690–0.962) for I.F. and 0.857 (95% CI, 0.664–0.876) for T.D.

### 2.3 Questionnaire

The questions were divided into two groups regarding demographics and dental history. Sex, age, and educational level were recorded. Dental hygiene habits were assessed as follows: brushing duration, frequency, and toothbrush type. The history of orthodontic therapy and smoking status were also evaluated (Table 1).

**TABLE 1:** Variables assessed in the questionnaire

Demographic variables
Age
Gender
Educational level (Junior high school, High school, University)
Dental history variables
Toothbrush bristles stiffness, classified as hard, medium and soft
Brushing duration (<1 min, 1–3 min, >3 min)
History of orthodontic treatment
Smoking assessment
No smokers
Light smokers (<10 cig/day)
Heavy smokers (≥10 cig/day)

### 2.4 Ethical considerations

The study protocol was approved by the Ethical Committee of the relevant institution (protocol number, 38/16‐05‐2016). All participants received written information regarding the aim and the procedures of the study, and afterwards, they were asked to sign their consent.

### 2.5 Statistical analysis

The sample size was estimated using the scenario considering a prevalence of 50% for any oral condition recorded. The expected frequency was 63.9%, based on a prevalence study conducted on the Greek population (Chrysanthakopoulos, 2014). It was also estimated that the multistage sampling used in the present study would yield approximately 50% inefficiency. Type I error was set at 0.05 level and power at 0.80. The sample size was calculated to be in total of 100 patients, using G*Power3.1. Descriptive statistics were calculated using the mean and standard deviation (SD) for continuous variables, frequencies, and percentages for categorical variables. Data normality was evaluated using the Kolmogorov–Smirnov test. A Mann–Whitney *U* test was applied to assess the relationship between gingival recession and different continuous variables: age, plaque index, bleeding, and GT. Chi^2^ test or Fisher's exact test was used to examine the association between gingival recession and different categorical variables: gender, education, periodontal health status, biotype, toothbrush type, brushing duration, brushing frequency, orthodontic therapy, and smoking status. Statistical analysis was performed using the STATA 13.0 software. The statistical significance level was set at a *p* value ≤ 0.05.

## 3 RESULTS

### 3.1 Demographic characteristics and clinical variables of the participants

The study group comprised 51 males and 53 females with a mean age of 23.6 ± 5.4 years (Range 18–30, Median 22.0). The mean % of the plaque index and GBI was 26.3 ± 17.1 and 22.9 ± 18.1, respectively. The demographics and clinical status parameters of the participants are presented in Table 2.

**TABLE 2:** Demographic characteristics and clinical variables of the included participants

Parameters	N (%)
Gender	
Male	51 (49.00)
Female	53 (51.00)
Age, mean (SD)	23.64 (5.38)
Education	
Junior high school	3 (2.80)
High school	9 (8.70)
University	92 (88.50)
Plaque index %, mean (SD)	26.3 (17.10)
Gingival bleeding index %, mean (SD)	22.9 (18.10)
Biotype	
Thin	34 (32.70)
Thick	70 (67.30)
Width of keratinized gingiva, median mean (SD)	1.2 (0.41)
Toothbrush type	
Soft	49 (47.10)
Medium	49 (47.10)
Hard	6 (5.80)
Brushing duration	
1 min	29 (27.90)
1–3 min	67 (64.40)
>3 min	8 (7.70)
Brushing frequency	
1 time/day	24 (23.1)
2 times/day	68 (65.4)
3 times/day	12 (11.5)
Orthodontic therapy	
No	59 (56.70)
Yes	45 (44.30)
Smoking status	
No	78 (75.00)
Light smokers	15 (14.40)
Heavy smokers	11 (10.60)

### 3.2 Prevalence of gingival recession

**FIGURE 1:**
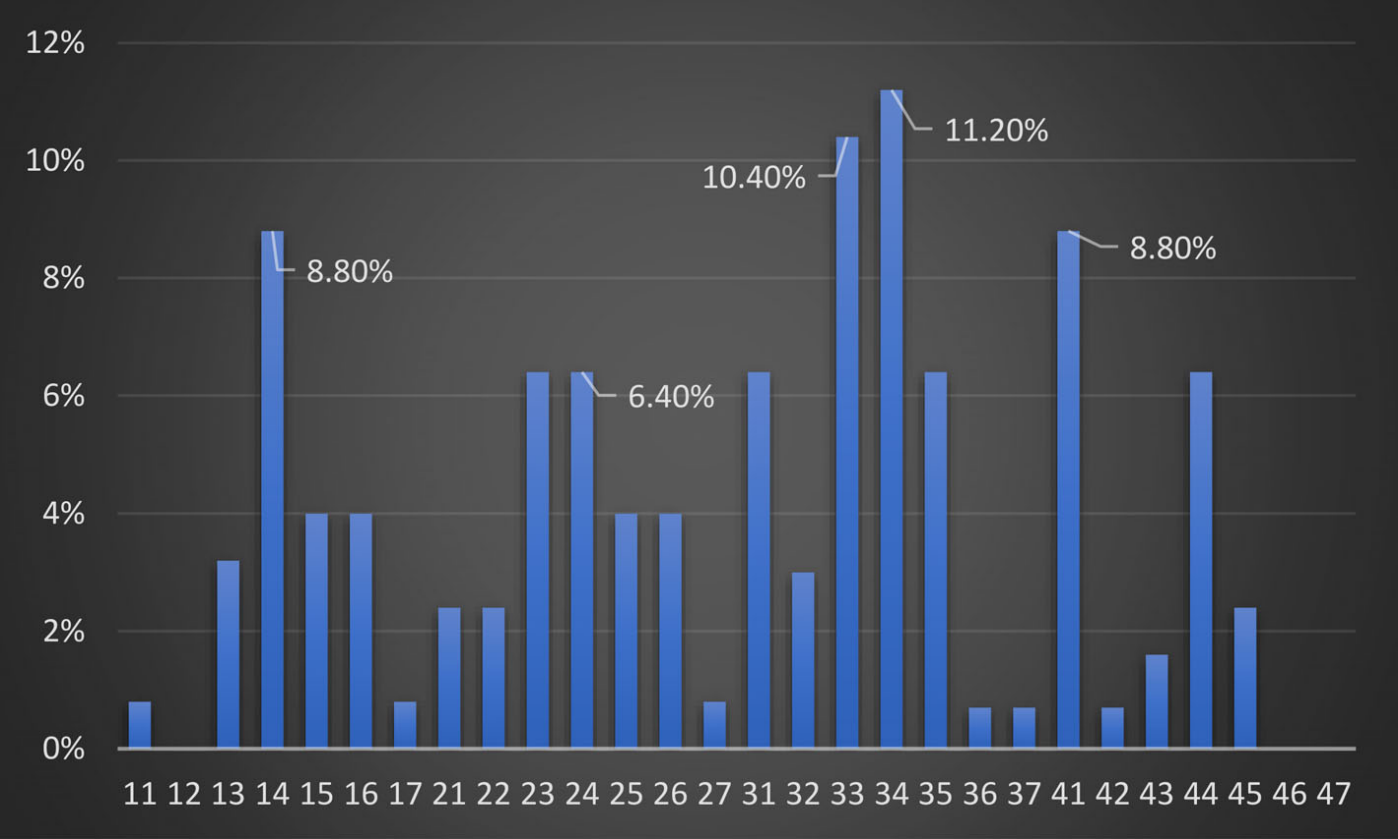
Distribution of gingival recession among tooth type

The presence of a gingival recession (at least one tooth with denuded root surface >1.0 mm) was found in 56/104 individuals (53.8%). This prevalence was slightly higher in males (29/51, 51.8%) than in females (27/53, 48.3%), but this difference was not statistically significant (Fischer's Exact test, *P* = 0.562). Among a total of 2912 examined teeth, there were 124 teeth (4.3%) with gingival recession. Furthermore, teeth most frequently associated with gingival recession were the left lower first premolar (11.2%) and the left mandibular canine (10.4%), followed by maxillary first premolars (8.8%) (Figure 1). Patients with gingival recession showed mean measurements of denuded root surface of a mean 1.43 ± 0.53 mm in both maxilla and mandible. Most of the teeth affected had a measurement of REC of 1 mm (67%) (Table 3).

**TABLE 3:** Distribution of RECs based of recession depth

Recession depth	*n*	%
1 mm	83	66.93
2 mm	28	22.58
3 mm	9	7.25
4 mm	3	2.41
5 mm	1	0.08

Distribution of RECs based of recession depth

### 3.3 Predisposing factors associated with gingival recession

#### 3.3.1 Gingival biotype

A thick biotype with a GT >1 mm was more prevalent (66.7%) in the population of the study. However, a statistically significant correlation between the gingival biotype and gingival recession could not be found as the prevalence of gingival recession was equally distributed between the two groups. (Fischer's Exact Test, *P* = 0.403) (Table 4).

**TABLE 4:** Associations of REC with the examined variables (* Variables associated with REC p<0.5)

Parameters	Gingival recession		*p* value
	No (*N* = 48) *N* (%)	Yes (*N* = 56) *N* (%)	
Gender			
Male	22 (45.80)	29 (51.80)	0.562
Female	26 (54.20)	27 (48.20)	
Age, mean (SD)	23.02 (5.24)	24.18 (5.68)	0.070
Education			
Junior high school	2 (4.25)	1 (1.9)	0.667
High school	6 (12.75)	5 (9.40)	
University	39 (83.00)	47 (88.70)	
Plaque index %, mean (SD)	24.83 (17.99)	27.56 (16.45)	0.182
Bleeding %, mean (SD)	18.84 (18.08)	26.17 (18.91)	0.024*
Biotype			
Thin	18 (37.50)	16 (28.60)	0.403
Thick	30 (62.50)	40 (71.40)	
Gingival thickness, mean (SD)	1.16 (0.39)	1.24 (0.43)	0.435
Toothbrush type			
Soft	22 (45.80)	27 (48.20)	0.273
Medium	25 (52.10)	24 (42.90)	
Hard	1 (2.10)	5 (8.90)	
Brushing duration			
1 min	18 (37.50)	11 (19.60)	0.086
1–3 min	28 (58.30)	39 (69.60)	
>3 min	2 (4.20)	6 (10.70)	
Brushing frequency			
1 time/day	12 (25.00)	12 (21.40)	0.240
2 times/day	28 (58.30)	40 (71.40)	
3 times/day	8 (16.70)	4 (7.20)	
Orthodontic therapy			
No	29 (60.40)	30 (53.60)	0.553
Yes	19 (39.60)	26 (46.40)	
Smoking status			
No	38 (79.20)	40 (71.40)	0.245
<10 cigarettes	4 (8.30)	11 (19.60)	
≥10 cigarettes	6 (12.50)	5 (9.00)	

#### 3.3.2 Other predisposing factors

Among the several factors examined, a statistically significant correlation could be found between REC and GBI. (Chi‐Square Test, *p* = 0.024). None of the other factors seemed to be associated with REC, although a slight tendency for a higher prevalence of GR was found in patients using a hard toothbrush or brushing for >3 min (Table 4).

## 4 DISCUSSION

The presence of gingival recessions is a common characteristic in young adult groups. In the current study, the prevalence of REC reached a value of 53.8%, which is consistent with the reported incidence of other similar studies (Checchi et al., 1999; Müller et al., 2002). A prevalence of 64% was reported in an Italian study including 55 Dental students (Checchi et al., 1999), while 50% of young adults aged 19–30 years old were presented with REC in a following study (Müller et al., 2002). In contrast, larger‐scale studies have reported a prevalence of 58–99.7% (Albandar & Kingman, 1999; Sarfati et al., 2010; Susin et al., 2004; Toker & Ozdemir, 2009). The difference between those studies and the present one might lie in the sample size and the average age of the participants. In a longitudinal study on REC, a mean prevalence of 7% was reported for the young age cohort and reached 19% after 12 years (Serino et al., 1994). In the study of Sarfati and coworkers, a mean prevalence of 84.6% was reported, however, the mean age of the participants was 49 years old. In addition, when age is taken into consideration gingival recessions due to periodontal disease become a more frequent phenomenon (Hegab & Alnawawy, 2020). Consequently, age is significantly associated with the prevalence of REC with older individuals having a higher probability of REC presence. (Albandar & Kingman, 1999; Löe et al., 1992; Rios et al., 2014; Susin et al., 2004). In our study, the presence of REC was not influenced by the age of the participant due to the strict age limit posed in the inclusion criteria. Upon comparison of our results with other studies reporting on similar age cohorts, the findings are quite intriguing. In the study of Checchi et al, REC reached 64%, while in a larger Brazilian cohort study the prevalence among the 20–29 age cohort was 76.5%. In contrast, an earlier study of Greek citizens indicated that 53.5% of the participants were affected by at least one REC, however, the percentage dropped to 15.6% in the youngest cohort (18–29 years old) (Chrysanthakopoulos, 2014). Similar results were obtained from a study in an Indian population were the mean prevalence of REC was 40.9% and dropped to 29.6% in the age cohort 25–35 (Mythri et al., 2015). Those discrepancies among studies can partially be explained by the geographic distribution of the examined population, including variable socio‐economic and educational status (Hegab & Alnawawy, 2020; Löe et al., 1992).

Considering the prevalence of REC at tooth level presenting 4.3%, it seems to be in accordance with the one reported in previous studies (2.5%–10.6%) in the same age cohort (Chrysanthakopoulos, 2014; Susin et al., 2004). Furthermore, 25% of the examined subjects harbored 77% of gingival recessions, a finding which agrees with a similar study where 19% of the included participants had 50% of the measured recessions (Serino et al., 1994). In other words, most gingival recessions were accumulated in a small number of subjects. The teeth most frequently implicated in REC were the mandibular left first premolar and the left mandibular canine, followed by maxillary first premolars, in accordance with previous studies, the teeth that were most often affected were canines and premolars. (Gorman, 1967). The results are also in accordance with the studies of Vignoletti et al. and Checchi et al. where the first premolars of all quadrants where the teeth with the most RECs (Checchi et al., 1999; Vignoletti et al., 2020). Nevertheless, other studies have found the lower central incisors to be the most frequently affected teeth (Albandar & Kingman, 1999; Susin et al., 2004). However, in those studies, the mean age of the participants is higher than in the current one. Previous studies have shown that the REC prevalence changes with progressing age, making mandibular incisors the most often affected teeth in older ages (Löe et al., 1992; Serino et al., 1994). Studies indicated that frequent and forceful tooth brushing, as well as toothbrushes with hard filaments, were associated with REC (Litonjua et al., 2003). In addition, premolars and canines were primarily affected, suggesting tooth profiles protrusion and position may contribute to recession. The analysis of the results showed a weak but negative association between toothbrushing duration and occurrence of REC as 75% of the individuals that brushed their teeth for more than 3 min showed REC. This finding was in accordance with recent data, which suggest that a positive association between excessive toothbrushing and REC is inconclusive (Jepsen et al., 2018; Rajapakse et al., 2007). This fact may explain the negative association between gingival recession and toothbrushing frequency or toothbrush type found in this study. A clear association could not be confirmed in the case of previous orthodontic treatment either. Although it consists a risk factor for REC development, factors such as the direction of the movement during brushing, or the GT may be more important than the treatment per se (Jepsen et al., 2018).

A thin gingival biotype has been considered as a significant predisposing factor for REC development (Cortellini & Bissada, 2018; Moawia & Cohen, 2003). In the current study, however, an association between REC and the biotype could not be confirmed. Although several clinical studies have proven the significant association of a thin biotype with the presence of REC (Di Jing et al., 2019; Liu et al., 2017), other studies have failed to do so (Chen et al., 2020; Shah et al., 2015). This can be attributed to the age of the participants or the biotype discrimination method. The method chosen in the present study for the characterization gingival biotype was the one proposed by Kan et al. (2010). The accuracy of the process lies in the fact that GT is a prerequisite measurement for the identification of periodontal biotype (Zweers et al., 2014). The method using probe visibility through the sulcus, although providing a higher sensitivity percentage, in our opinion cannot define the actual GT (De Rouck et al., 2009). The choice of a different biotype discrimination technique would potentially offer a more transparent relationship between the examined parameters. Besides, recent data suggest the presence of three distinct biotypes, a fact that was not considered in the present study (Cortellini & Bissada, 2018).

Among the other predisposing factors examined the only factor associated with REC presence of gingival inflammation assessed by the GBI. In the current study, both patients with gingivitis and periodontally healthy were included in order to address the prevalence of REC in this young age group. Previous studies have confirmed the role of gingival inflammation in recession initiation (Müller et al., 2002; Sarfati et al., 2010). In addition, the role of persistent gingival inflammation in the establishment of REC has been suggested in histological studies (Baker & Seymour, 1976). Of interest was the finding that a negative association between plaque scores and REC was found, as the role of poor oral hygiene in the development of gingival recession is established (Cortellini & Bissada, 2018; Hegab & Alnawawy, 2020; Moawia & Cohen, 2003). This was not the case in our study where the sample was quite homogenous in terms of oral hygiene standards as 69% of the participants had a plaque score <30%. Several studies have suggested a positive association between high educational level and occurrence of gingival recession (Chrysanthakopoulos, 2014; Susin et al., 2004). The presence of such an association could be attributed to the fact that more educated individuals recognize the value and importance of oral hygiene and more effectivily comply with it rendering the presence of recession more frequent. In our study, such an association could not be proved due to the high educational level of the majority of the participants.

The last factor examined was the influence of cigarette smoking. Nor in this case, a positive association could be identified. Previous studies have acknowledged the effects of tobacco smoking in the development of REC (Sarfati et al., 2010; Susin et al., 2004). However, a 6‐month follow‐up study in a group of young individuals failed to demonstrate that smokers had an increased risk for REC or REC progression (Müller et al., 2002). Despite the conflicting evidence on the impact of smoking in the presence of REC, a clear relationship has not been established.

We must mention some limitations of the study design. The limited sample size may have underestimated the existence of some associations among REC and predisposing factors; however, power analysis reached a high percentage (80%), enabling accurate final analysis of the sample. The method chosen for gingival biotype assessment may be debatable, concerning the accurate determination of biotype clusters, though it is an established technique applied by a number of clinical studies investigating this parameter which have confirmed the accuracy and repeatability of this method in daily clinical practice (Fischer et al., 2015; Zweers et al., 2014). Lastly, the cross‐sectional design of the study could not allow for an observational effect of several factors on REC over time; However, it can be concluded that the prevalence of REC is relatively high among a small young Greek cohort, indicating the need for a raise of awareness among clinicians for the preventive management and treatment of REC.

## AUTHOR CONTRIBUTIONS

Loannis Fragkioudakis designed the study, performed the clinical examinations, wrote and revised the draft and performed the statistical analysis. Dimitra Tassou aided the clinical sample collection and organised the data. Maria sideri was responsible for patient inclusion and distribution. Ioannis Vouros, conseptualised the study design, supervised the study and revised and checked the drafts.

## Data Availability

The data used to support the findings of this study are available from the corresponding author upon request.
